# Supplementary material for: Wide-range continuous tuning of the thermal conductivity of La0.5Sr0.5CoO3-δ films via room-temperature ion-gel gating
Source: Nat Commun. 2023 May 6;14:2626. doi: 10.1038/s41467-023-38312-z (PMC10164146; doi:10.1038/s41467-023-38312-z)
Supplement: Supplementary file 1 — Supplementary Information [file 41467_2023_38312_MOESM1_ESM.pdf]

Supplementary Information for:

Wide-range continuous tuning of the thermal conductivity of $La_{0.5}Sr_{0.5}CoO_{3-\delta}$ films *via* room-temperature ion-gel gating

Yingying Zhang[1], William M. Postiglione[2], Rui Xie[3], Chi Zhang[1], Hao Zhou[3], Vipul Chaturvedi[2], Kei Heltemes[2], Hua Zhou[4], Tianli Feng[3], Chris Leighton[2*] and Xiaojia Wang[1*]

[1]*Department of Mechanical Engineering, University of Minnesota, Minneapolis, MN55455, USA*

[2]*Department of Chemical Engineering and Materials Science, University of Minnesota, Minneapolis, MN55455, USA*

[3]*Department of Mechanical Engineering, University of Utah, Salt Lake City, Utah 84112, USA*

[4]*Advanced Photon Source, Argonne National Laboratory, Lemont, Illinois 60439, USA*

**Authors to whom correspondence should be addressed. Electronic mail: leighton@umn.edu; wang4940@umn.edu*

1. Structural characteristics of LSCO films

Details for samples measured in this study are summarized in Table S1, including the channel area, film thickness, gate voltage, crystalline phase, and out-of-plane lattice parameter.

Table S1. Summary of film information for XRD and TDTR measurements corresponding to Fig. 1c and Fig. 3a. *Asterisks indicate films for which XRD data were not taken, and therefore phase composition is inferred based on electronic transport, TDTR, and XRD from other samples.

Sample #	Channel area (mm^2)	Film thickness (nm)	Gate voltage (V)	Phase	*c*-axis lattice parameter (Å)
1	1 × 1	45	0.0	P	3.88
2	1 × 1	45	1.0	P	3.89
3	1 × 1	45	1.4	P + BM*	---
4	1 × 1	45	1.8	P + BM*	---
5	1 × 1	45	2.0	P + BM	3.94
6	1 × 1	45	2.5	P + BM*	---
7	1 × 1	45	2.6	P + BM*	---
8	1 × 1	45	3.0	BM	3.99
9	1 × 1	58	4.0	BM	4.00
10	4 × 3.5	45	3.5	BM	3.99

In addition to the structural characterization shown in Fig. 1c of the main text, further characterization of as-grown perovskite-phase $La_{0.5}Sr_{0.5}CoO_{3-\delta}$ (P-LSCO) films were performed *via* wide-angle specular XRD using synchrotron radiation (λ = 0.5636 Å) at the Advanced Photon Source, Argonne National Lab, and reciprocal space mapping (RSM) using in-house Cu Kα radiation (see Methods). Figure S1a shows the synchrotron XRD pattern collected around the substrate LAO(002) reflection for an as-grown P-LSCO film (4 × 3.5 mm^2), this film being representative of other LSCO films investigated in this work. The visible Laue oscillations in Figure S1a indicate low surface and substrate/interface roughnesses, the oscillation periodic spacing indicating a thickness of ~45 nm. The P-LSCO (002) peak was fit to a Gaussian, and the extracted full-width-at-half-maximum produced a coherence length comparable to the film thickness (~35 nm), further indicative of high crystalline quality. Figure S1b additionally shows the reciprocal space map (RSM) of a similar film about the LAO(103) asymmetric reflection. The P-LSCO film intensity is centered along the vertical line H = 1, indicating that the film has the same in-plane lattice parameter as the substrate, *i.e.*, it is fully strained, with some minor strain relaxation evidenced by the broad, low-intensity region skewed toward the relaxed position. Note that the (103) Bragg reflection peak for the LAO substrate is relatively broad due to the twin domains prevalent in commercial LAO substrates.

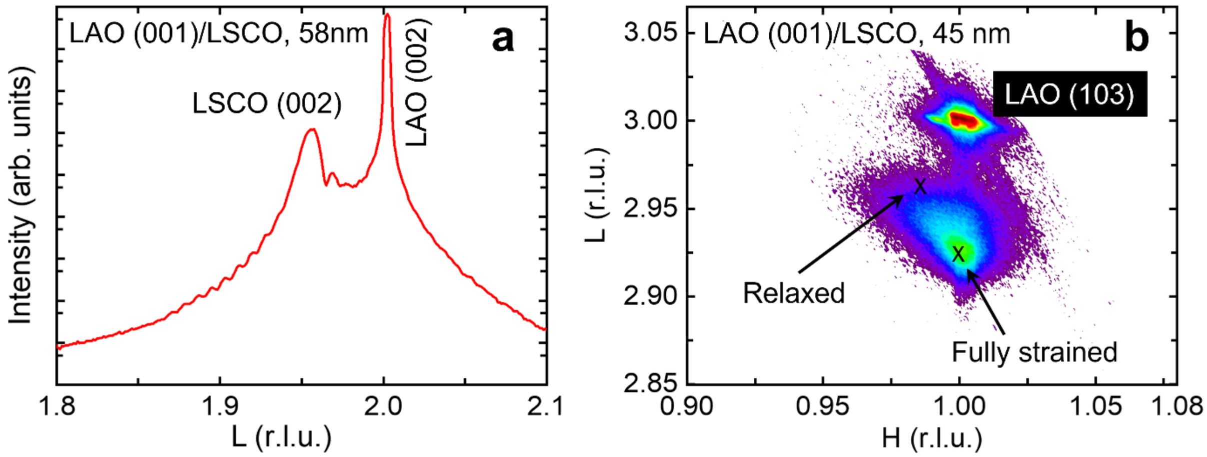

Fig. S1 | Wide-angle specular X-ray diffraction characterization of as-grown P-LSCO films sputter-deposited on LAO(001) substrates. **a** Specular (002) synchrotron X-ray diffraction (λ = 0.5636 Å) peaks of the 45-nm P-LSCO film and substrate. **b** Reciprocal space map taken around the LSCO film and LAO substrate (103) peaks. The ×'s mark the positions of the film peak expected for the fully strained (pseudomorphic) and relaxed positions, respectively. "r.l.u." (reciprocal lattice units) are with respect to the LAO substrate (a = 3.79 Å).

XRD characterization was also performed on gated films, as shown in Fig. 1c in the main article. Scans on the samples gated to 1, 2, and 3 V have relatively low signal-to-noise ratio, especially around the emerging BM peaks (006) and (0010), due to the small film area (1 × 1 mm^2) in this case. To more carefully probe the BM structure, a larger-channel-area device (4 × 3.5 mm^2) was also gated to 3.5 V, and the XRD results are shown in Fig. 1c (ungated (black) and 3.5 V (magenta)). The more optimized geometry in this sample reduces the intensity of the 'ungated' perovskite (*i.e.*, the film buried beneath the Pt electrode), reduces the intensity of the Pt(111) peak (from the Pt electrodes), and significantly increases the signal-to-noise ratio for the LSCO film peaks, which is especially important for the lower-intensity BM (006) and (0010) reflections. This improved signal-to-noise ratio allows more quantitative analysis, where the full-width-at-half-maximum (FWHM) was estimated by using Gaussian peak fitting, for example. While the positions of the BM (006) and (0010) peaks gave the same out-of-plane lattice parameter as the (008) (~4.0 Å), suggesting all peaks indeed derive from the same phase, the (006) and (0010) are

significantly broader (larger FWHM), resulting in lower out-of-plane coherence lengths (as calculated from the Scherrer formula). The extracted coherence length was found to be ~10 – 15 nm for the (006) and (0010) *versus* 25 – 30 nm for the (008), indicating substantial disorder in the BM phase. Additionally, the observed intensity ratio between BM (008) and (006) (or (0010)) was ~70, while the ratio expected for fully ordered BM is ~10 – 40, as found in various reports of high-quality *as-grown* BM $SrCoO_{2.5}$ films[1-6]. This larger intensity ratio is consistent with prior work for ion-gel-gate-induced BM-LSCO[7], and is likely due to the presence of additional defects which disrupt the long range V_O ordering. Based on the above observations, we thus conclude that the BM-LSCO formed here *via* ion-gel-gating, particularly the anion (oxygen) sublattice, features significant additional disorder compared to as-grown BM SCO films.

2. Electrolyte gating: *in-situ* two-terminal resistance *vs.* gate voltage

Ion-gel gating of 45-58 nm LSCO films was carried out at 300 K under vacuum ($< 1 \times 10^{-5}$ Torr) by sweeping the gate voltage (V_g) at a rate of 0.5 mV sec^{-1} to the target voltage (see Methods). *In-situ* electronic transport measurements were taken by applying a source-drain voltage ($V_{SD} = \pm$ 0.2 V) throughout the experiment, measuring the source-drain current, and determining the source-drain resistance, R_{SD}, as a function of V_g. Figure S2 shows the R_{SD} *vs.* V_g measured for the 11 1 × 1 mm channel films in Table S1. Gating starts at $V_g = 0$, sweeping to larger positive V_g at 0.5 mV sec^{-1} and stopping at specific V_g values as needed for *ex situ* characterization (XRD, resistivity, and TDTR). The films start as perovskite (P) (R_{SD} ~ 200 Ω) and gradually increase in resistance with increasing V_g until the transformation to brownmillerite begins. Then a rapid increase in R_{SD} occurs on entry into the phase coexistence region (P + BM) around 2 – 3 V. This is followed by another relatively flat region ($R_{SD} \sim 10^5$ Ω) indicating fully formed BM. Overall, the ion-gel-gated

samples show good repeatability, with only minor sample-to-sample variations, around the transition region (2 – 3 V) and at the highest V_g (4 V). Note that the 4 × 3.5 mm channel sample is not shown here due to the larger resistance owing to its larger channel length. Finally, after the conclusion of each gating experiment, the ion gels were removed, the films cleaned with acetone, and *ex-situ* four-wire van der Pauw resistance measurements were performed (see Fig. 1d & Fig. 3a in the main article).

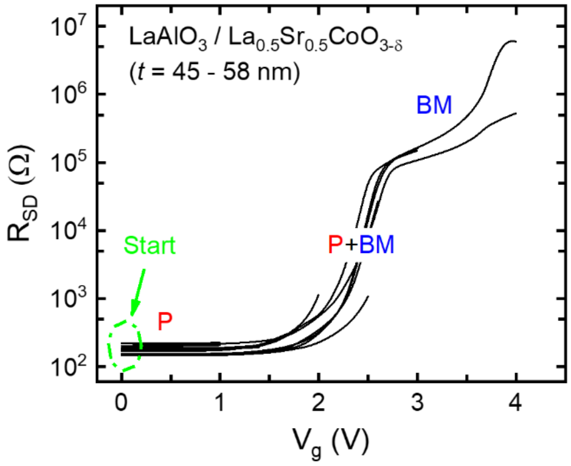

Fig. S2 | Two-terminal resistance. Source-drain two-terminal resistance (R_{SD}) *vs.* gate voltage (V_g) for 11 1 × 1 mm channel area ion-gel-gated LSCO films of 45-58 nm thickness on LAO(001) substrates. Gating was performed at 300 K under vacuum.

3. Interfacial thermal conductance of Pt/LSCO and LSCO/LAO interfaces

In TDTR measurements, only parameters with sufficient sensitivities can be uniquely determined. The measurement sensitivity to a certain parameter is calculated as[8]:

$$S_\alpha = \frac{\partial \ln R}{\partial \ln \alpha}, \tag{1}$$

where α is the parameter of interest, S_α is the measurement sensitivity to the parameter α, and R is the ratio signal from TDTR. In TDTR measurements, heat propagation into the sample is treated as thermal waves at the modulation frequency f. Subsequently, the thermal penetration depth of

the thermal wave (h) can be calculated based on $h = \sqrt{\frac{\Lambda}{\pi C f}}$, where C is the heat capacity and Λ is the thermal conductivity of the sample material. For thermal measurements of thin films, when h is smaller than the film thickness d, the film is thermally opaque. The TDTR signal is then not sensitive to the substrate and the interfacial thermal conductance of the interface between the film and the substrate (G_2). On the contrary, when h is larger than d, the film becomes thermally thin. In this case, the TDTR signal becomes sensitive to the properties of the LAO substrate (Λ_{LAO} and C_{LAO}) and G_2. Due to the ultra-thin film nature of our LSCO samples, h is larger than the film thickness for all three modulation frequencies used in TDTR measurements (1.5, 9.0, and 18.8 MHz). Therefore, measurement conditions must be carefully designed to distinguish the sensitivities to Λ_{LSCO}, G_1, and G_2 for reliable determination of these parameters.

Here, we take the as-grown P-LSCO sample (ungated) as an example to show how we design the experiments and data reductions to extract Λ_{LSCO}, G_1, and G_2 with reasonable accuracy. Figure S3a-c shows the routine TDTR measurement sensitivity to multiple parameters under different modulation frequencies for the ungated sample. It is found that routine TDTR measurements with three modulation frequencies have the highest sensitivities to the properties of the Pt transducer and LAO substrate. Therefore, prior to measuring the LSCO films, we obtained the properties of the Pt transducer and LAO substrate by measuring reference samples of Pt on Si/SiO_2(300 nm) and Pt on LAO prepared from the same sputtering batch. These properties of the transducer and substrate were used as input parameters for data reduction of LSCO thin-film measurements to improve the reliability. For routine TDTR, the measurement sensitivity is more reasonable for Λ_{LSCO} than G_1 or G_2. In this case, we extracted Λ_{LSCO} from routine TDTR measurements by simultaneously fitting all three modulation frequencies. For determination of G_2, since routine TDTR measurements have low sensitivity to G_2, the dual-frequency TDTR approach

was adopted to increase the sensitivity to G_2[9]. As illustrated in Fig. S3d, the sensitivities of dual-frequency TDTR measurements (18.8 MHz/1.5 MHz) to the Pt transducer and LAO substrate are largely suppressed, while the sensitivity to G_2 is improved. Using the dual-frequency approach, G_2 was determined to be 800 ± 500 MW m^{-2} K^{-1}, which agrees with the literature values for strongly bonded interfaces[10]. The ~60% uncertainty on G_2 does not propagate significant errors into the overall uncertainty of Λ_{LSCO}. This is because the routine TDTR measurement sensitivity to G_2 is much lower than the measurement sensitivity to Λ_{LSCO}.

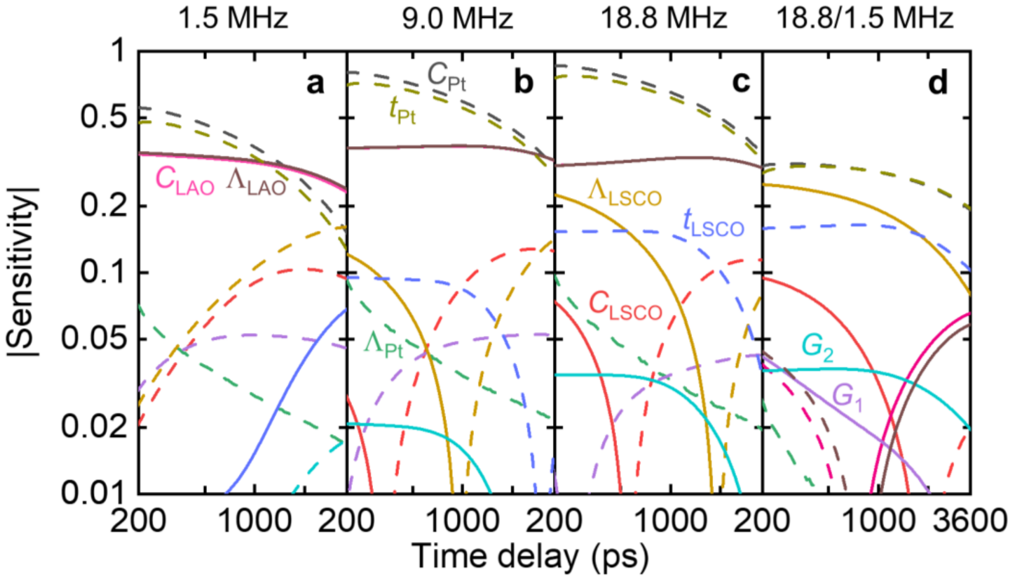

Fig. S3 | Representative sensitivity analyses. Sensitivity plots of TDTR measurements for the ungated P-LSCO sample calculated with the following parameters: heat capacities (C_{Pt}, C_{LSCO}, C_{LAO}), film thicknesses (t_{Pt}, t_{LSCO}), thermal conductivities (Λ_{Pt}, Λ_{LSCO}, Λ_{LAO}), interfacial thermal conductances (G_1 and G_2), and the beam spot size of 12 μm. Panels **a-c** are routine TDTR ratio sensitivity to multiple parameters with different modulation frequencies. Panel **d** is the dual-frequency measurement sensitivity. Solid lines mean the sensitivity value is positive, while dashed lines represent negative sensitivity values.

Also from Fig. S3, the measurement sensitivity to G_1 is low in all cases due to the large value of G_1 of the ungated sample, leading to large measurement uncertainties. This is expected considering the extra thermal transport channel at the Pt/P-LSCO interface through electron-

electron coupling. When G_1 decreases as P-LSCO (metallic) transforms to BM-LSCO (insulating) with electrolyte gating, the measurement sensitivity to G_1 is improved, which allows for the extraction of G_1 for samples gated at higher voltages (V_g > 2 V, see Fig. 3b). To validate our measured values of G_1 for BM-LSCO films exhibiting insulting behavior, we list G values for interfaces between Pt and different insulating oxides[11-14] in Fig. S4, which vary from 180 to 330 MW m^{-2} K^{-1}. Our measurement results for G_1 between Pt and BM-LSCO samples (gated at 3, 3.5 and 4 V with negligible electronic contribution) are determined to be 160, 150 and 200 MW m^{-2} K^{-1}, respectively, in good agreement with literature data.

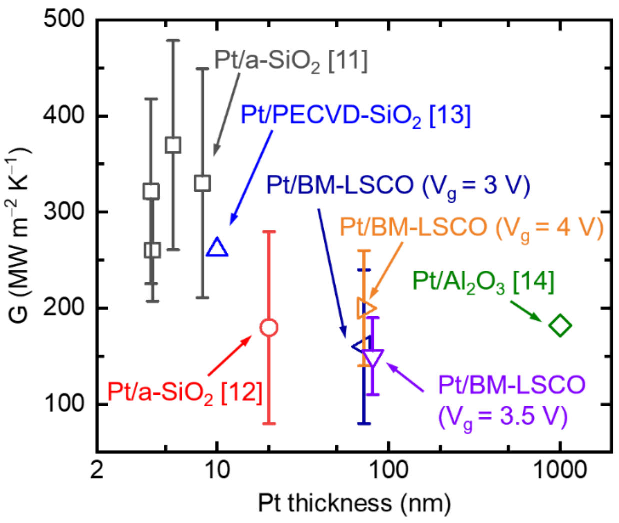

Fig. S4 | Interfacial thermal conductance. Summary of the interfacial thermal conductance between Pt and oxides plotted against the Pt thickness. The G values of Pt/SiO_2 and Pt/Al_2O_3 are taken from the literature[11-14].

For samples gated at $V_g \leq 2$ V, with larger G_1 values, a nominal value of 400 MW m^{-2} K^{-1} was used for the data reduction. Due to the low sensitivity to G_1, the determination of Λ_{LSCO} is not significantly impacted by G_1. Table S2 summaries the values of G_1 and Λ_{LSCO} from measurement fitting. When both parameters are fitted simultaneously, G_1 varies from 250 to 920 MW m^{-2} K^{-1},

while Λ_{LSCO} only varies from 4.1 to 5.5 W m^{-1} K^{-1}. When G_1 is fixed at 400 MW m^{-2} K^{-1}, the fitted Λ_{LSCO} (listed in the last column of Table S2) varies from 4.1 to 6.3 W m^{-2} K^{-1}. The variations of Λ_{LSCO} with or without G_1 being fixed in the data analysis are all within the measurement uncertainty.

Table S2. Results of G_1 and Λ_{LSCO} for the ungated samples measured at different locations and modulation frequencies, obtained from simultaneous fitting for both G_1 and Λ_{LSCO} or fitting for Λ_{LSCO} only (G_1 = 400 MW m^{-2} K^{-1}).

**Sample #**	**Location #**	**Modulation frequency (MHz)**	**G_1 (MW m^{-2} K^{-1})**	**Λ_{LSCO} (W m^{-1} K^{-1})**	**G_1 (MW m^{-2} K^{-1})**	**Λ_{LSCO} (W m^{-1} K^{-1})**
		1.5	920	4.7		6.3
	L1	9	490	4.3		4.3
		18.8	240	5.0		5.1
		1.5	280	5.5		4.5
**1**	L2	9	530	4.2		4.1
		18.8	320	4.6		4.7
		1.5	390	4.6		4.5
	L3	9	600	4.5	400	4.3
		18.8	410	4.1		4.1
		1.5	520	4.3		4.9
	L1	9	660	4.1		4.3
**2**		18	330	4.9		5.0
		1.5	440	4.7		4.9
	L2	9	250	4.9		4.4
		18	440	4.5		4.5
**Average (range)**			455 (240 - 920)	4.6 (4.1 – 5.5)	400 (fixed)	4.7 (4.1 – 6.3)

In addition, we followed the thickness-extrapolation method used by Wu *et al.* for quantifying the lumped thermal resistances of two interfaces ($R_{eff} = 1/\Lambda_{LSCO} + 1/G_1 + 1/G_2$)[15], as a criterion to cross check the reasonableness of our measured G_1 and G_2. We prepared ungated LSCO films with varying thicknesses and coated them with Pt. The lumped thermal resistances R_{eff} of Pt/P-LSCO measured with TDTR are represented in Table S3 as a function of the LSCO film thickness. The linear extrapolation of R_{eff} to zero film thickness (intercept on the *y*-axis) gives $1/G_1$

+ $1/G_2$ = 6.15 nm m K W^{-1} with ~50% uncertainty. With G_1 = 400 MW m^{-2} K^{-1} and G_2 = 800 MW m^{-2} K^{-1}, the calculated R_{eff} value (3.75 nm m K W^{-1}) is within the uncertainty.

Table S3. R_{eff} of ungated LSCO films with varying thicknesses on LAO substrates.

Film thickness (nm)	Lumped thermal resistance R_{eff} (nm m K W^{-1})
5.2	6.42 ± 2.24
10.7	9.17 ± 2.75
15.6	9.36 ± 2.81
19.8	8.59 ± 2.83

4. Uncertainty analysis for TDTR measurements

For TDTR measurements, the uncertainty on LSCO thermal conductivity is calculated based on the sensitivity analysis using the following expression:

$$\left(\frac{\Delta\Lambda}{\Lambda}\right)^2 = \sum\left(\frac{S_\alpha}{S_\Lambda}\frac{\Delta\alpha}{\alpha}\right)^2 + \left(\frac{S_\phi}{S_\Lambda}\,\delta\phi\right)^2, \tag{2}$$

where $\Delta\Lambda/\Lambda$ is the relative uncertainty of the sample thermal conductivity Λ, $\Delta\alpha/\alpha$ is the relative uncertainty of a certain parameter α, $\delta\phi$ is the uncertainty of the phase, and S_α is the measurement sensitivity to a parameter α. With a combination of a 3% uncertainty in the beam spot size, a 4% uncertainty in the transducer thickness, a 3% uncertainty in the heat capacities of the transducer, sample, and substrate, and an 11% uncertainty in the substrate thermal conductivity, the resulting uncertainty on the ungated P-LSCO thermal conductivity is ~43%. The main contributions to the total uncertainty on the LSCO thermal conductivity are from Λ_{LAO}, C_{LAO}, d_{Pt} and C_{Pt}. As Λ_{LSCO} decreases when V_g increases, the measurement sensitivity to Λ_{LSCO} is also improved. As shown in Fig. S5, the measurement sensitivity to Λ_{LSCO} for the BM-LSCO sample gated at 3 V significantly increases compared to the ungated P-LSCO sample. Therefore, the uncertainty of the 3-V-gated LSCO is reduced to ~10%.

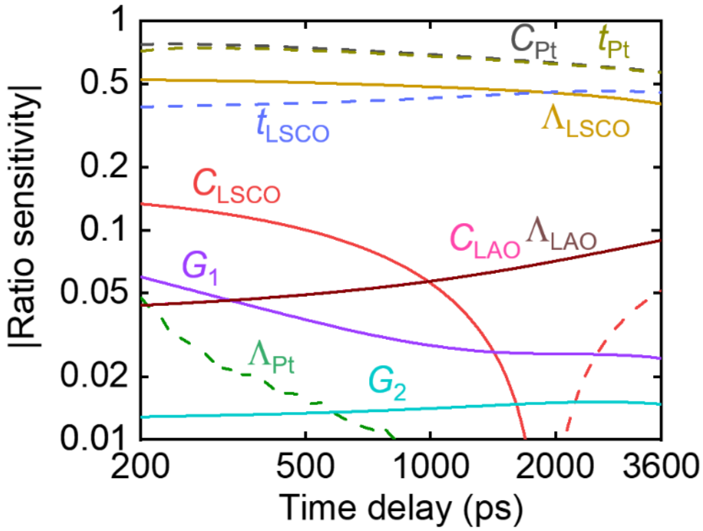

Fig. S5 | Sensitivity plot for uncertainty analyses. Sensitivity plot for routine TDTR measurements at 18.8 MHz for the LSCO sample gated at 3 V for uncertainty calculations.

5. MD simulations of LSCO thermal conductivity

To study the effect of oxygen defects on thermal transport in LSCO, we theoretically predicted the lattice thermal conductivity of LSCO with different oxygen deficiencies (δ = 0.1, 0.25, 0.375, and 0.5) using molecular dynamics (MD) simulations (Fig. 3d) with the LAMMPS package. The potentials used in LAMMPS in this work are the short-range Buckingham potential + long-range Coulombic potential, which are two-body potentials. Therefore, we expect that the heat flux formalism in LAMMPS should be reliable. Classical potentials are not as accurate as first-principles calculations, nor can they correctly predict optical branches. However, our goal here is to provide qualitative insights to better interpret the experimental results, rather than pursuing very high accuracy in BTE calculations. The equilibrium Green-Kubo method was used to calculate the thermal conductivity of LSCO:

$$k_i = \frac{1}{k_B T^2 V} \int_0^\infty < S_i(t) \cdot S_i(0) > dt, i = x, y, z, \quad (3)$$

where S is the transient heat flux, and $<S_i(t) \cdot S_i(0)>$ is the heat flux autocorrelation function, which measures the correlation between the heat flux at any time with itself after time t. k_B is the Boltzmann constant, T is temperature, and V is the volume of the simulation domain. The domain was constructed by using 12 × 12 × 12 unit cells (8640 atoms) of $LaCoO_3$ and replacing half the La atoms with Sr. Oxygen atoms were removed to create point vacancy defects either randomly or periodically depending on the needs. Our algorithm ensures the vacancies are point defects instead of cluster defects. The Buckingham and Coulomb interatomic potential was used to describe the interactions[16]. The timestep was set as 0.5 fs. We first relaxed the geometries at constant pressure and temperature (NPT ensemble) at 2 K for 0.2 ns (400000 steps) and then increased the temperature to 300 K and ran for another 0.2 ns. Then, the ensemble was changed to constant volume and energy (NVE ensemble) and run for 0.2 ns to stabilize the ensemble. Finally, the NVE ensemble was run for another 2 ns to compute the heat flux and auto-correlation function. For each individual structure, the thermal conductivity was obtained by averaging the thermal conductivities of three independently generated structures with randomly distributed V_O along the x, y, and z directions. In addition, for the case of $\delta = 0.5$, we generated two sets of structures: disordered V_O and ordered V_O, with the latter case being the orthorhombic BM phase.

To validate the equilibrium GKMD results, we further performed non-equilibrium molecular dynamics (NEMD) for four cases with the non-stoichiometry δ values that have been studied with EMD (*i.e.*, $\delta = 0.1$, 0.25, 0.375, and 0.5). The NEMD simulation domain is shown in Fig. S6a with a size of 2.2938 × 3.0584 × 3.0584 nm^3. NEMD simulations were performed using the LAMMPS package with the same classical potential settings as implemented in the GKMD. The hot reservoir was set in the middle of the simulation domain with a length of 2 nm, and the cold reservoir of the same size was split into two pieces equally and placed at the two edges of the

box along the x direction. In all the three dimensions, periodic boundary conditions were applied. A Langevin thermostat was adopted to maintain the temperatures of the hot reservoir at 598.15 K and the cold reservoir at 548.15 K. The timestep was set as 0.5 fs. In the simulation, the NVT ensemble was fixed first for 100,000 steps and then followed by an NVE ensemble for 100,000 steps to fully relax the lattice. Then the system was fixed at the NVE ensemble for another 200,000 steps, during which the temperature gradient and heat flow rate were recorded. Then the thermal conductivity can be obtained based on Fourier's law. Figure S6b shows an example of the temperature gradient along the x-axis for the $\delta = 0.25$ case. It is noticed that the hot reservoir has the highest temperature, while the cold reservoir exhibits the lowest temperature. The length between the hot and cold reservoir is 9.2 nm, different from that in Fig. S6a. This discrepancy is caused by the position where the temperature is monitored not being precisely at the boundary of the reservoirs. Figure S6c displaces the cumulative heat flow applied to or extracted from the system. Linear fitting is applied to obtain the heat flow rate. The heat flow rate and temperature difference on both sides are averaged when evaluating the thermal conductivity.

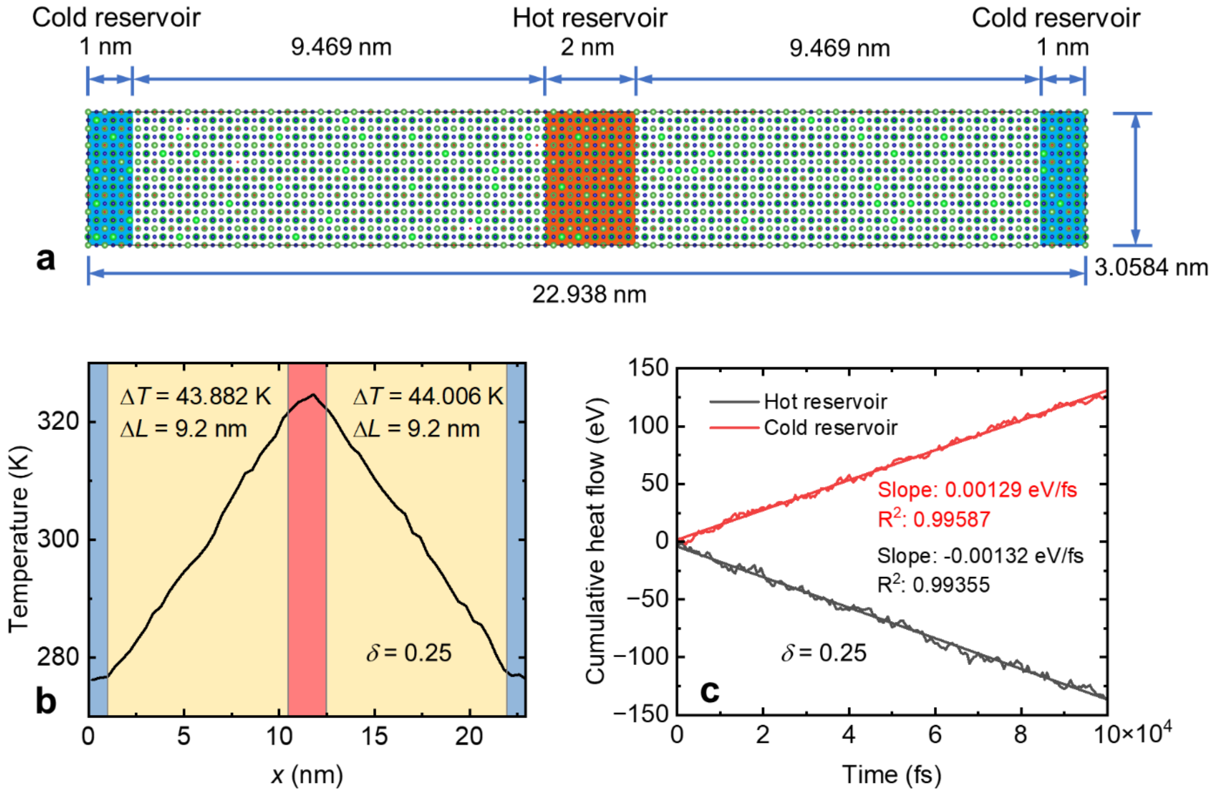

Fig. S6 | NEMD setup and results. **a** Dimensions of the NEMD simulation system, and the hot and cold reservoirs in the *x*-*y* plane. **b** Temperature gradient along the *x* direction for the $\delta = 0.25$ case. **c** Cumulative thermal energy in the hot and cold reservoirs.

6. Temperature-dependent thermal measurements

For data analysis of temperature-dependent thermal measurements, we need to obtain the temperature-dependent volumetric heat capacities of the Pt transducer, the LSCO film, and the LAO substrate (C_{Pt}, C_{LSCO}, C_{LAO}), and the temperature-dependent thermal conductivities of Pt and LAO (Λ_{Pt} and Λ_{LAO}) as input parameters. C_{Pt} and Λ_{Pt} were obtained from the literature[17-19]. C_{LAO} and C_{LSCO} were calculated using the Debye model following:

$$C = 5 \times \frac{9\bar{R}\rho}{M} \left(\frac{T}{\theta_D}\right)^3 \int_0^{\frac{\theta_D}{T}} \frac{x^4 e^x \, dx}{(e^x - 1)^2}, \tag{4}$$

where R is the universal gas constant (8.314 J mol^{-1} K^{-1}), ρ is the mass density, M is the molar mass, T is temperature, and θ_D is the Debye temperature (748 K for LAO[19] and 462 K for LSCO[20]).

The calculated values of temperature-dependent C_{LAO} and C_{LSCO} are presented in Fig. S7a in comparison with literature data[19,21-23].

We measured a bare LAO substrate with the same Pt transducer to obtain the temperature-dependent thermal conductivity of LAO. For thermal conductivity measurements at low T, temperature correction is necessary considering that material properties may vary significantly over a small temperature rise. The temperature correction takes into account two sources of temperature rise: (1) the steady-state temperature rise (ΔT_s), and (2) the per-pulse pump heating induced temperature rise (ΔT_p). The calculation of ΔT_s can be found in Ref. [24]. ΔT_p was calculated as $\Delta T_p = Q/(VC_{Pt})$, where Q is the pump energy absorbed by the Pt transducer ($Q = 2a_{Pt}A_{Pump}/f_{laser}$, with a_{Pt} being the absorptivity of Pt, A_{pump} being the absorbed pump power, and f_{laser} being the repetition rate of the laser), V is the volume of a cylinder (with a $1/e^2$ radius of the pump beam spot size and height of the Pt thickness), and C_{Pt} is the volumetric heat capacity of Pt. The corrected temperature was calculated as $T_{corr} = T_{set} + \Delta T_p + \Delta T_s$ with T_{set} being the setting temperature set in the temperature controller. Those input parameters were then modified based on T_{corr} for a new round of data reduction. We iterated this correction process several times until we obtained converged results (*e.g.*, $\Delta T_p + \Delta T_s \leq 15$ K).

Figure S7b shows the comparison between our measured Λ_{LAO} and literature values of Λ_{LAO} along different crystalline orientations from experimental studies[19,21-23,25]. Due to the existence of twin boundaries in LAO, the thermal conductivity of LAO along the [100] direction is much lower than that along the [001] direction (black symbols) and the temperature-dependent thermal conductivity deviates from the $1/T$ trend. Our measured temperature-dependent Λ_{LAO} along the [100] direction agrees reasonably well with literature data. The measured Λ_{LAO} values were then used as input parameters for LSCO measurements at different temperatures.

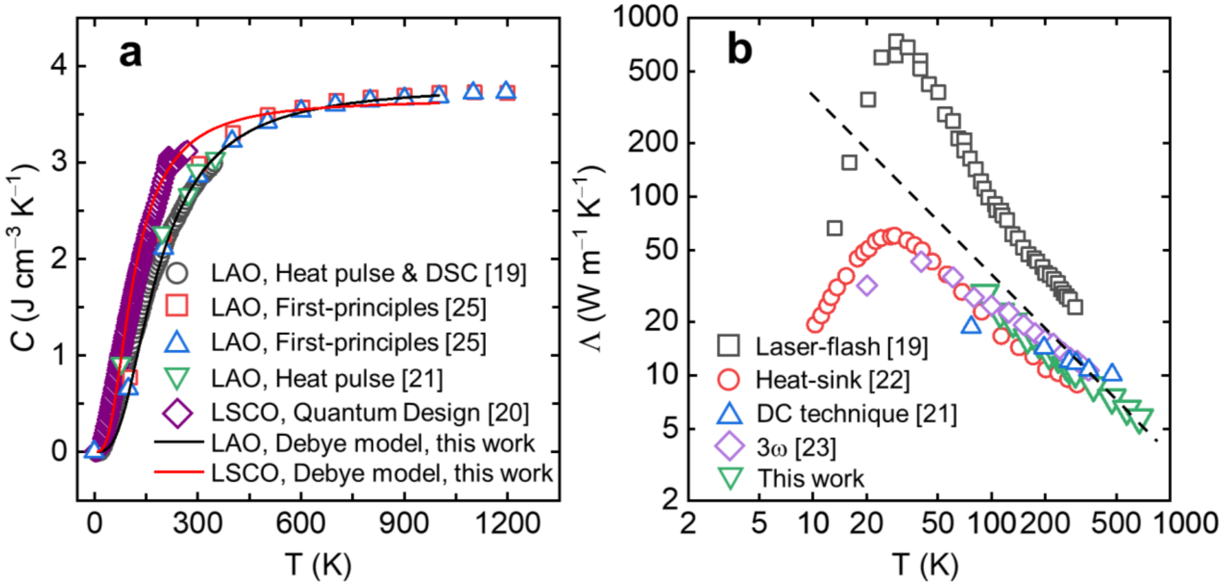

Fig. S7 | *T*-dependent heat capacity and thermal conductivity. **a** *T*-dependent heat capacity of LAO[19,21-23] and LSCO[20]. Note that in Ref. [20], x = 0.3 is the highest x studied for single-crystal $La_{1-x}Sr_xCoO_3$, and is thus the closest sample system to our $La_{1-x}Sr_xCoO_3$ (x = 0.5) film available in the literature. **b** *T*-dependent thermal conductivities of LAO obtained from different measurement methods[19,21-23,25]. The black dashed line indicates the 1/T trend of temperature-dependent thermal conductivity.

In addition to the BM-LSCO film gated to 3 V, we also conducted temperature-dependent thermal measurements of the BM-LSCO film gated at 4 V and the results are plotted in Fig. S8. The thermal conductivity of the sample gated to 4 V (red diamonds) agrees well with that of the sample gated to 3 V (blue circles) over the measurement temperature range of ~ 90 to 500 K. This further supports, from the thermal characterization aspect, our finding that LSCO is fully transformed to the BM phase after being gated at 3 V and above.

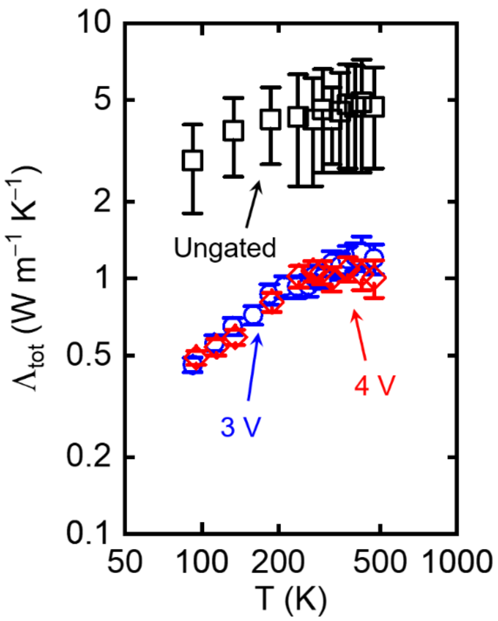

Fig. S8 | *T*-dependent thermal conductivity. Temperature-dependent total thermal conductivities of LSCO films at different gate voltages.

7. Comparison of in-plane vs. through-plane transport properties

Due to the low measurement sensitivity to the in-plane thermal conductivity of LSCO thin films at these types of thicknesses[26], in addition to the challenges of probing the through-plane electrical resistivity, we were unable to measure the electronic and thermal transport along the same direction. However, considering that the thickness of our measured samples is ~45 nm, we can safely conclude that the electronic transport in our P-LSCO samples exhibits bulk behavior. Estimates of the electronic mean free path in this system yield nanometric scales. Also, from *ab initio* calculations of P-$SrCoO_3$ in a prior study[27], the accumulated lattice thermal conductivity of P-$SrCoO_3$ is larger than 93% of its bulk value when the phonon mean free path reaches 40 nm. In our P-LSCO system, with the additional alloying on the A site, the lattice thermal conductivity will approach the bulk limit at an even smaller film thickness. In addition, considering the cubic symmetry of P-LSCO, we expect quite isotropic band structures for both electrons and phonons.

Thus, the through-plane thermal conductivity of P-LSCO, consisting of both electronic and lattice contributions, must be comparable to the in-plane thermal conductivity.

Unlike P-LSCO, BM-LSCO is electrically insulating; therefore, thermal transport in BM-LSCO is mainly carried by phonons and will also approach the bulk limit. However, there is possible anisotropy for thermal transport in BM-LSCO due to the orthorhombic symmetry of the BM phase. In this case, the large tuning factor of the LSCO thermal conductivity we demonstrated in this work is along the through-plane (*c*-axis) direction.

8. Reversibility of tuning the LSCO thermal conductivity

To explore the reversible nature of the V_g-induced P $\leftrightarrow$ BM phase transformation in LSCO, we also reverted some gated BM-LSCO films to the P phase by applying reverse gate voltages of up to –4.5 V at RT. The RT thermal conductivity of one reverse-gated P-LSCO film (P $\rightarrow$ BM $\rightarrow$ P) was measured to be 3.5 ± 1.1 W m^{-1} K^{-1}. This ~20% reduction in the Λ_{tot} of recovered P-LSCO compared to the as-deposited P-LSCO film arises from additional structural disorder induced during the cyclic gating. This can be seen from the high-resolution XRD measurements (Fig. S9a,b) on the as-deposited P-LSCO film (labeled as "ungated"), after gating to +3.0 V (*i.e.*, transformed to BM), and after reverse gating to –4.5 V (*i.e.*, recovered to P). The ideal situation after negative V_g would be a P(002) reflection exactly overlapping with the ungated peak. In reality, the return to the ungated P(002) location from the BM(008) position has a remnant shift to the left in Fig. S9b (*i.e.*, a larger P lattice parameter). Explicitly, the BM(006) and (0010) peaks are entirely extinguished, definitively establishing that long-range V_O order is annihilated, but the lattice parameter of the recovered P phase remains expanded. This indicates that the V_O concentration is higher than it was originally for the ungated sample. This additional structural disorder can be

further quantified through electronic transport (Fig. S9c). Specifically, the approach described in the main text and Methods of our paper to determine the O non-stoichiometry δ from transport yields 0.11 for the as-deposited P-LSCO film and 0.16 for the reverse-gated film (following P → BM → P). This additional disorder is unsurprising after topotactic transformation from P to BM back to P, and no doubt plays some role in the reduced thermal conductivity. Nevertheless, such data confirm the overall reversibility of the approach presented here. Further improvement of reversibility can be achieved through better device design and optimized gating conditions.

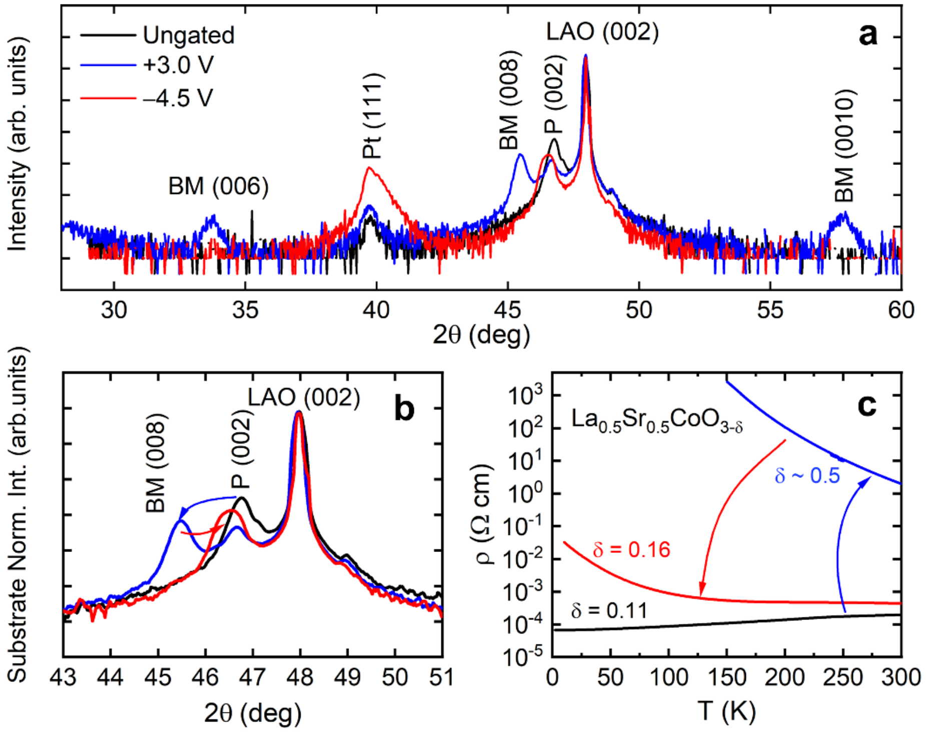

Fig. S9 | XRD measurements on a reverse-gated sample. a Wide-angular-range XRD results from LSCO devices on LAO(001) substrates after gating to +3.0 V and after reverse gating to –4.5 V. The *y*-axis is on a logarithmic scale. **b** An enlarged version of the same scans as in a, around the film and substrate perovskite (002) peak (BM (008)), with the intensity axis (log scale) normalized to the substrate intensity, to better show the changes in the film position and intensity with reverse gating. **c** Temperature-dependent electrical resistivity measurements of the as-deposited P-LSCO, after gating to +3.0 V, and after reverse gating to –4.5 V (the color code is the same for all panels).

References

1 Lu, N. *et al.* Electric-field control of tri-state phase transformation with a selective dual-ion switch. *Nature* **546**, 124-128 (2017).

2 Lu, Q. & Yildiz, B. Voltage-controlled topotactic phase transition in thin-film $SrCoO_x$ monitored by in situ x-ray diffraction. *Nano Lett.* **16**, 1186-1193 (2016).

3 Katase, T., Suzuki, Y. & Ohta, H. Reversibly switchable electromagnetic device with leakage-free electrolyte. *Adv. Electron. Mater.* **2**, 1600044 (2016).

4 Hu, S., Wang, Y., Cazorla, C. & Seidel, J. Strain-enhanced oxygen dynamics and redox reversibility in topotactic $SrCoO_{3-\delta}$ ($0< \delta \leq 0.5$). *Chem. Mater.* **29**, 708-717 (2017).

5 Cui, B. *et al.* Electric-field control of oxygen vacancies and magnetic phase transition in a cobaltite/manganite bilayer. *Phys. Rev. Appl.* **8**, 044007 (2017).

6 Jeen, H. *et al.* Reversible redox reactions in an epitaxially stabilized $SrCoO_x$ oxygen sponge. *Nat. Mater.* **12**, 1057-1063 (2013).

7 Chaturvedi, V. *et al.* Doping-and strain-dependent electrolyte-gate-induced perovskite to brownmillerite transformation in epitaxial $La_{1-x}Sr_xCoO_{3-\delta}$ Films. *ACS Appl. Mater. Interfaces* **13**, 51205-51217 (2021).

8 Zhu, J., Wu, X., Lattery, D. M., Zheng, W. & Wang, X. The ultrafast laser pump-probe technique for thermal characterization of materials with micro/nanostructures. *Nanosc. Microsc. Therm.* **21**, 177-198 (2017).

9 Jiang, P., Huang, B. & Koh, Y. K. Accurate measurements of cross-plane thermal conductivity of thin films by dual-frequency time-domain thermoreflectance (TDTR). *Rev. Sci. Instrum.* **87**, 075101 (2016).

10 Wilson, R., Apgar, B. A., Hsieh, W.-P., Martin, L. W. & Cahill, D. G. Thermal conductance of strongly bonded metal-oxide interfaces. *Phys. Rev. B* **91**, 115414 (2015).

11 Kimling, J., Philippi-Kobs, A., Jacobsohn, J., Oepen, H. P. & Cahill, D. G. Thermal conductance of interfaces with amorphous SiO_2 measured by time-resolved magneto-optic Kerr-effect thermometry. *Phys. Rev. B* **95**, 184305 (2017).

12 Liu, J., Choi, G.-M. & Cahill, D. G. Measurement of the anisotropic thermal conductivity of molybdenum disulfide by the time-resolved magneto-optic Kerr effect. *J. Appl. Phys.* **116**, 233107 (2014).

13 Chien, H.-C., Yao, D.-J. & Hsu, C.-T. Measurement and evaluation of the interfacial thermal resistance between a metal and a dielectric. *Appl. Phys. Lett.* **93**, 231910 (2008).

14 Hopkins, P. E., Norris, P. M. & Stevens, R. J. Influence of inelastic scattering at metal-dielectric interfaces. *J. Heat Transf.* **130**, 022401 (2008).

15 Wu, X. *et al.* Glass-like through-plane thermal conductivity induced by oxygen vacancies in nanoscale epitaxial $La_{0.5}Sr_{0.5}CoO_{3-\delta}$. *Adv. Funct. Mater.* **27**, 1704233 (2017).

16 Read, M. S., Islam, M. S., Watson, G. W., King, F. & Hancock, F. E. Defect chemistry and surface properties of $LaCoO_3$. *J. Mater. Chem.* **10**, 2298-2305 (2000).

17 Yokokawa, H. & Takahashi, Y. Laser-flash calorimetry II. Heat capacity of platinum from 80 to 1000 K and its revised thermodynamic functions. *J. Chem. Thermodyn.* **11**, 411-420 (1979).

18 Powell, R., Tye, R. & Woodman Margaret, J. Thermal conductivities and electrical resistivities of the platinum metals. *Platin. Met. Rev.* **6**, 138-143 (1962).

19 Schnelle, W., Fischer, R. & Gmelin, E. Specific heat capacity and thermal conductivity of $NdGaO_3$ and $LaAlO_3$ single crystals at low temperatures. *J. Phys. D* **34**, 846 (2001).

20 He, C. *et al.* Heat capacity study of magnetoelectronic phase separation in $La_{1-x}Sr_xCoO_3$ single crystals. *Phys. Rev. B* **80**, 214411 (2009).
21 Michael, P. C., Trefny, J. U. & Yarar, B. Thermal transport properties of single crystal lanthanum aluminate. *J. Appl. Phys.* **72**, 107-109 (1992).
22 Morelli, D. T. Thermal conductivity of high temperature superconductor substrate materials: Lanthanum aluminate and neodymium aluminate. *J. Mater. Res.* **7**, 2492-2494 (1992).
23 Langenberg, E. *et al.* Analysis of the temperature dependence of the thermal conductivity of insulating single crystal oxides. *APL Mater.* **4**, 104815 (2016).
24 Cahill, D. G. Analysis of heat flow in layered structures for time-domain thermoreflectance. *Rev. Sci. Instrum*. **75**, 5119-5122 (2004).
25 Boudali, A., Amrani, B., Abada, A. & Amara, K. First-principles study of structural, elastic, electronic, and thermal properties of $LaAlO_3$ perovskite. *Comput. Mater. Sci.* **45**, 1068-1072 (2009).
26 Zhu, J. *et al.* Revealing the origins of 3D anisotropic thermal conductivities of black phosphorus. *Adv. Electron. Mater.* **2**, 1600040 (2016).
27 Lu, Q. *et al.* Bi-directional tuning of thermal transport in $SrCoO_x$ with electrochemically induced phase transitions. *Nat. Mater.* **19**, 655-662 (2020).
